# GBA-associated Parkinson’s disease in Hungary: clinical features and genetic insights

**DOI:** 10.1007/s10072-023-07213-w

**Published:** 2023-12-28

**Authors:** Tamás Szlepák, Annabel P. Kossev, Dóra Csabán, Anett Illés, Szabolcs Udvari, Péter Balicza, Beáta Borsos, Annamária Takáts, Péter Klivényi, Mária J. Molnár

**Affiliations:** 1 Institute of Genomic Medicine and Rare Disorders, Semmelweis University, Budapest, Hungary; 2 HUN-REN, Multiomic Neurodegeneration Research Group, Budapest, Hungary; 3 Department of Internal Medicine and Oncology, Semmelweis University, Budapest, Hungary; 4 Department of Neurology, Semmelweis University, Budapest, Hungary; 5 Department of Neurology, Faculty of Medicine, Albert Szent-Györgyi Clinical Center, University of Szeged, Szeged, Hungary

**Keywords:** GBA-associated Parkinson’s disease, GBA1 mutation, Phenotype-genotype correlation, Genetic risk factor

## Abstract

**Introduction:**

Parkinson’s disease (PD) has a complex genetic background involving both rare and common genetic variants. Although a small percentage of cases show a clear Mendelian inheritance pattern, it is much more relevant to identify patients who present with a complex genetic profile of risk variants with different severity. The ß-glucocerebrosidase coding gene (*GBA1*) is recognized as the most frequent genetic risk factor for PD and Lewy body dementia, irrespective of reduction of the enzyme activity due to genetic variants.

**Methods:**

In a selected cohort of 190 Hungarian patients with clinical signs of PD and suspected genetic risk, we performed the genetic testing of the *GBA1* gene. As other genetic hits can modify clinical features, we also screened for additional rare variants in other neurodegenerative genes and assessed the APOE-ε genotype of the patients.

**Results:**

In our cohort, we identified 29 *GBA1* rare variant (RV) carriers. Out of the six different detected RVs, the highly debated E365K and T408M variants are composed of the majority of them (22 out of 32). Three patients carried two *GBA1* variants, and an additional three patients carried rare variants in other neurodegenerative genes (*SMPD1*, *SPG11*, and *SNCA*). We did not observe differences in age at onset or other clinical features of the patients carrying two *GBA1* variants or patients carrying heterozygous APOE-ε4 allele.

**Conclusion:**

We need further studies to better understand the drivers of clinical differences in these patients, as this could have important therapeutic implications.

**Supplementary Information:**

The online version contains supplementary material available at 10.1007/s10072-023-07213-w.

## Introduction

Parkinson’s disease is a complex neurodegenerative disorder with an increasingly intricate genetic background. While well-known monogenic forms with Mendelian inheritance exist, emerging evidence highlights the importance of oligogenic and polygenic effects, as well as the potential pathogenic role of endolysosomal or autophagosomal pathways and rare variants of genes in these pathways [1–5]. Pathogenic mutations of ß-glucocerebrosidase enzyme coding gene (GBA1) lead to Gaucher disease (GD) when inherited in the biallelic pattern. However, inherited in a monoallelic state, they are the largest genetic risk factor for developing PD [6], with an approximate frequency of 2–25% in the clinically diagnosed PD patients. The wide range in frequency is mainly attributed not only to the difference in genetic background of various ethnicities, but also to the fact that many original studies focused only on specific mutations, mainly those which are associated with GD [7]. There are three types of GD, distinguishable based on the presence of neurological symptoms [8], but there is only a very mild overlap between the neurological phenotype of GD and PD. Additionally, the majority of GD patients do not develop PD. Thus, the current view is that decreased enzyme activity is not the sole cause of PD development. This is consistent with reports of various enzyme activity levels in PD patients with or without *GBA1* rare mutations [9] and the lack of correlation between activity and the severity or progression of clinical symptoms [10].

The heterozygous mutations in the *GBA1* gene and subsequent impairment of enzyme function seem to contribute to alpha-synuclein (a-syn) aggregation, which in reverse affect intracellular trafficking of GCase, creating a pathological loop [11]. The effects of heterozygous *GBA1* variants can be seen in neuroimaging studies [12],  as well as the increased number of identified *GBA1* variant carriers among PD and DLB cases examined by autopsy [13, 14].

*GBA1*-associated PD phenotypically can be characterized mostly as sporadic PD but is usually associated with an earlier age of onset, while cognitive decline and psychiatric symptoms can be dominating signs as well [15]. Furthermore, motor complications such as dysphagia, also non-motor complications, such as REM (rapid eye movement) sleep disorders have also been reported more frequently in *GBA1* carriers [16].

There is growing evidence of gene-gene interactions in *GBA*-associated PD. Observational studies described the earlier onset of PD symptoms for patients carrying both LRRK2 G2019S and *GBA1* variant [17, 18], while others found inhibition of LRRK2 kinase can normalize the detrimental effect of reduced GCase enzymatic activity in *GBA1*-mutated astrocytes or iPSC-derived neurons [19]. In one of the biggest aggregated PD-focused datasets, researchers found that Parkinson’s disease and dementia with Lewy bodies (DLB) patients with *GBA1* variants often also carry a substantial number of other PD-associated risk variants, which can modify the age of onset [20].

This is in alignment with earlier studies showing the genetic burden of variants in other lysosomal storage disorder genes in PD cases, reinforcing the importance of lysosomal mechanisms in the development of PD [21]. The relevance of exploring second genetic hits can be seen in families identified with familial PD, where multiple members of the family carried *GBA1* rare variants, but only those with a second rare variant showed symptoms of the disease [22].

Currently, the ε4 allele of APOE (*APOE-ε4*) is considered the strongest genetic risk factor for autosomal dominant early-onset AD and late-onset AD in a dose-dependent manner [23], but it was also found to be associated with DLB in GWAS [24]. In recent years, evidence is emerging showing that carriers of *APOE-ε4* alleles have accelerated decline of cognitive function and progression to Parkinson’s disease dementia or dementia in Parkinson’s disease [25–27], and also that there may be an interaction between the ApoE genotype and *GBA1* mutations in the development of PD [28, 29]. Szwedo AA et al. followed PD patients clinically for 10 years and reported that carriers of both *GBA1* and *APOE-ε4* alleles had faster cognitive decline and were at a higher risk for dementia [28]. A similar effect was reported by Shiner and her colleagues, on cognitive and motor impairment among DLB patients harboring the *APOE-ε4* allele and *GBA1* variants [29]. However, more research is needed to fully understand the potential interaction not only between these two genetic factors in PD but also between *GBA1* mutations and other genetic factors as well.

## Patients and methods

### Study cohort

Our study included patients whose data and samples were stored in the NEPSYBANK, the biobank of the Institute of Genomic Medicine and Rare Disorders. Written informed consent in accordance with the Declaration of Helsinki was signed by patients and control subjects before blood collection and molecular genetic analysis were made. The study was approved by the Hungarian Scientific and Research Ethical Committee. Molecular genetic analysis was performed for diagnostic purposes in all investigated patients.

We selected patients from our biobank who were considered to have either “clinically established PD” or “clinically probable PD” based on the Movement Disorder Society (MDS) Clinical Diagnostic Criteria for Parkinson’s Disease (*N*=190). We included PD patients with positive family history assuming genetic background (*N*=46), and PD cases having relatives with Gaucher disease (*N*=1). With two exceptions, all patients were of Hungarian descent. Subjects with pathogenic variants in established Mendelian Parkinson’s disease genes (*SNCA*, *LRRK2*, *PARK2*, *PARK7*, or *PINK1*) were excluded from the analysis.

All patients were examined by a board-certified neurologist. Some patients were referred to our center for genetic studies. Our cohort included 109 males and 81 females, with an average age of onset of 54.3 +12.7 years.

### Molecular genetic analysis

DNA was isolated from the patients’ peripheral blood samples using the QIAamp DNA blood kit, according to the manufacturer’s protocol (QIAgen, Hilden, Germany). The rare variants were identified either by Sanger sequencing (using ABI Prism 3500 DNA Sequencer, Applied Biosystems, Foster City, USA) of the entire *GBA1* gene coding regions, done from previous studies of our lab in 36 cases, by NGS in targeted panel sequencing (Illumina MiSeq) in 138 cases, or by whole exome sequencing (Illumina Hiseq) in 14 cases.

Molecular genetic testing was done as previously published [30]. By NGS-targeted panel sequencing, the following PD-associated genes were investigated: *PRKN(PARK2)*, *DJ1(PARK7)*, *PINK1*, *LRRK2*, *SNCA*, and *GBA1*. The whole list of tested genes by the targeted NGS panel can be seen in Supplementary file 1. In the case of Sanger sequencing, the *GBA1* gene was amplified using nested PCR in order to avoid the sequencing of its highly homologous pseudogene (*GBAP1*). In the application of short-read NGS sequencing, the presence of a pseudogene and the possibility of recombinant events between the pseudogene and the coding gene means a high chance of false positive findings, due to the use of unspecific primer pairs and small amplicons. Thus, Sanger sequencing as described above was performed to confirm all of the positive findings of NGS (targeted panel and WES). If the rare variant was already identified in a subject, only the specific exon was sequenced by Sanger sequencing in further family members. During analysis, the NM_001005742.2 reference sequence was used from the GeneBank dataset. The leader sequence (exon1) was not included in the procedure of analysis.

### ApoE testing

The *ApoE* genotype of patients was tested by the RFLP (restriction fragment length polymorphism) method according to the slightly modified protocol of Hixson JE and Vernier DT [31]. For the validation of allelic variant detection, Sanger sequencing was used.

### Bioinformatic analysis

The detailed protocol for bioinformatic analysis was described by our group in [30]. Briefly, the variant calling was done by GATK HaplotypeCaller, the SnpEff tool was used for annotation, and an in-house built software (VariantAnalyzer, Budapest University of Technology and Economics) was used for variant filtering and analysis. For the diagnosis, only non-synonymous rare variants were considered, whose frequency did not exceed 1% in the Genome Aggregation database (gnomAD v2.1).

## Results

### Variant frequencies

The coding region of the *GBA1* gene was sequenced in 188 patients who had Parkinsonian sign; additionally, specific exons of *GBA1* of two patients who were family members having PD symptoms. In this cohort, we detected six different types of *GBA1* monoallelic heterozygous rare variants in 29 people from 27 families indicating that these variants were not unique or specific for a family or individual person.

**Fig. 1:**
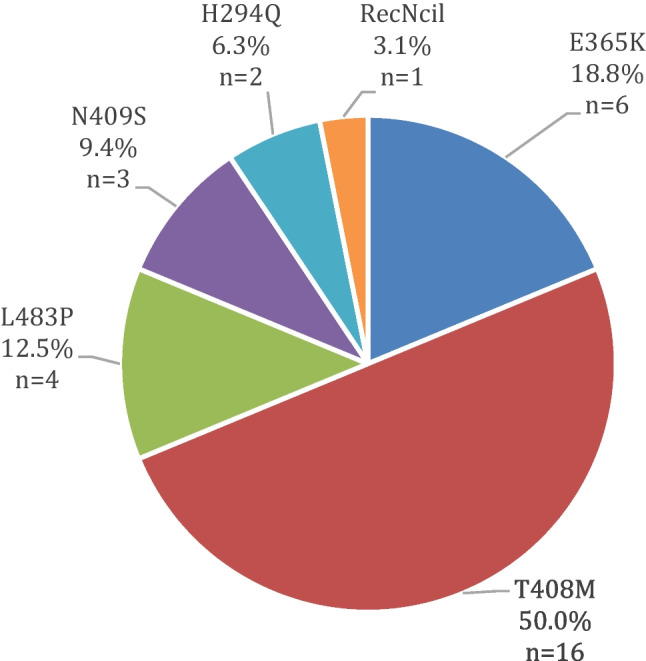
The percentages of the detected rare variants in our investigated cohort

In our cohort, the most frequent rare variants were T408M (*n*=16) and E365K (*n*=6). These two variants comprised 69% of the identified variants. The L483P was present in four cases, the N409S in three cases, the H294Q in two cases, and the RecNciI variant was carried by one patient (Fig. 1). Two patients were heterozygous both for T408M and E365K variants, and one patient was heterozygous for T408M and N409S variants. The detailed table of the variants, the mean age of onset (AOO), and the sex distribution are indicated in Table 1. The mean AOO of symptoms of patients with *GBA1* variants was 44.6 + 12.3 years. The male-female sex ratio was 21:8. More females had the E365K variant, than males in our cohort; however, this could be due to the small cohort size.

**Table 1:** Patient demographic data in association with the identified rare variants in the GBA1 gene

Patient ID	Variant HGVS	Mean AOO+SD	Sex
P1, P2, P4, P5, P6, P8, P9, **P10**, **P12**, P13, P14, P15, P21, **P23,** P25, P29	T408M	42.7 + 10.5	4♀ 12♂
P3, **P10**, P11, P20, **P23,** P27	E365K	50.6 + 4.5	5♀ 1♂
P18	RecNciI	61	1♂
P16, P24	H294Q	48 + 24	2♂
**P12**, P17, P26	N409S	40.5 + 0.7	3♂
P7, P19, P22, P28	L483P	42.5 + 9.8	4♂

*AOO* age of onset, *SD* standard deviation

Three of the patients (P7, P14, P16) carried GBA1 RV out of the 14 whole exomes sequenced, and we identified three patients (P10, P16, P25) who carried beside the *GBA1* rare variant an additional rare heterozygous variant in genes which are associated with PD based on OMIM or on the scientific literature.

Only the results of patients having *GBA1* variants are discussed in our current report.

### Clinical symptoms of GBA1 RV carriers

For the detailed characterization of patients’ phenotype, we focused on both motor and non-motor symptoms of PD.

At disease onset, motor symptoms were present in 75.8% (22/29) of patients, including tremor, bradykinesia, arm swing asymmetry, or clumsiness; tremor was the most frequent symptom, occurring in 17 cases. Overall, 31% (*n*=9) of patients showed signs of cognitive dysfunction, 62% (*n*=18) had psychiatric symptoms, and 31% (*n*=9) suffered from variable autonomic dysfunctions. During the course of the study, most patients received clinical care, which is why fluctuations in symptom severity were common.

The frequency of the non-motor symptoms that are considered common in *GBA1*-associated PD patients were the following in our cohort: hyposmia 17.2% (*n*=5), sleep disturbances 20.6% (*n*=6), and autonomic dysfunctions such as obstipation 13.8% (*n*=4), urogenital dysfunction 20.6% (*n*=6), and orthostatic hypotension 3% (*n*=1). Three patients (P23, P24, and P28) had episodic hallucinations (auditory and/or visual).

Carriers of the most frequent variants, the T408M and/or E365K (*n*=19) had an average subtype of motor symptoms of 4.3, the most frequent of these were asymmetric arm swing 73.6% (*n*=14), slowed fast alternating movements 57.8% (*n*=11), muscle stiffness 52.6% (*n*=10), and decreased fine motor movements or clumsiness 47.3% (*n*=9). In this group, the most common psychiatric symptoms were cognitive dysfunctions 26.3% (*n*=5), depression 21% (*n*=4), and apathy 21% (*n*=4), while the most frequent autonomic dysfunction was urogenital dysfunction 26.3% (*n*=5). A comparison of non-motor symptoms between E365K and/or T408M variants and other rare *GBA1* variant carriers can be seen in Table 2.

**Table 2:** Percentages of symptom groups of E365K and/or T408M carriers compared to carriers of H294Q, N409S, L483P, or RecNciI variants. P12 was excluded since he was a carrier of T408M and N409S variants

Patient characteristics	E365K and/or T408M	Other rare *GBA1* variants
Sex	8♀ 11♂	9♂
Mean AOO+SD	44.2 + 10.5	46 + 13
Cognitive dysfunction	31.5%	33.3%
Psychiatric symptoms	57.8%	66.6%
Autonomic dysfunction	31.5%	33.3%

*AOO* age of onset, *SD* standard deviation

In our cohort, P12, the carrier of T408M and N409S, showed the most severe and broad type of motor symptoms. In contrast to this, he had no psychiatric or autonomic dysfunctions, and his sole complaint was depression.

Patients with double *GBA1* heterozygous mutations (P10, P12, P23) showed no unique symptoms, earlier onset (47+6.2 compared to 44.3+11.5 years), or faster progression compared to the average of single heterozygous carriers. In regard to their genotype, the variants of P10 were in the *trans* position, but we do not have information about the cis/trans position of the variants in the other two subjects, since we were not able to investigate their family members.

Furthermore, we identified a coexisting rare variant besides the *GBA1* variants in three patients. One patient (P16) carried a rare variant in another lysosomal gene; he had the *SMPD1*: c.872G>A likely pathogenic missense variant. In a female patient (P25), the *SNCA* c.347T>C VUS missense variant coexisted. In P10—who had the *GBA1* E365K and T408M variants in trans position—the *SPG11*: c.5623C>T pathogenic nonsense mutation was detected as well.

### Comparison of APOE-ε genotypes of GBA1 RV carriers

We genotyped all 29 *GBA1* carriers for ApoE alleles (Table 3). We have found that four of our patients carried one *APOE*-ε2, 18 patients carried biallelic *APOE*-ε3, and seven had monoallelic *APOE*-ε4 (Table 3). No biallelic *APOE*-ε4 was detected in our cohort. There was no difference in the AOO of *APOE*-ε3 and *APOE*-ε4 allele carriers (45.7+8.8 vs 43.1+13.3). The *APOE*- ε4 allele did not show a significantly higher odds ratio in association with cognitive dysfunction OR 1.95 (95% CI= 0.32–12.01).

**Table 3:** Comparison of APOE-ε genotypes of GBA1 RV carriers regards their AOO and presence of cognitive dysfunction

ApoE genotypes	Heterozygous *APOE*-ε2	Homozygous *APOE*-ε3	Heterozygous *APOE*-ε4
Number of patients	4 (13.8%)	18 (62%)	7 (24.1%)
Mean AOO+SD	43 ± 17.7	45.7 ± 8.8	43.1 ± 13.3
Cognitive dysfunction	25% (*n*=1)	27.8% (*n*=5)	42.8% (*n*=3)

*AOO*, age of onset; *SD*, standard deviation

### Special cases with coexisting rare variants

#### Case 1

P10 is a female patient whose symptoms started at the age of 52 with clumsiness and reduced arm-swing symmetry of the left hand, pain in the left lower limb, and dizziness. Her brain MR was normal. She was diagnosed with early-onset PD and was treated accordingly. Six years later, her Parkinsonian symptoms progressed: she had daily freezing, and her “off” periods occasionally lasted hours. Neurological examination identified hyposmia, rigor, bradykinesia, micrographia, anxiety, and constipation. In her family, both parents had dementia at an old age and her maternal uncle had late-onset PD. Her daughters (2) were healthy, both were in their 30s. Genetic testing identified the E365K and T408M variants in the *GBA1* gene, as well as an early truncating stop rare variant in *SPG11*(NM_025137.4):c.5623C>T/p.Q1875*. All three variants were heterozygous. Segregation studies of her relatives identified the two *GBA1* variants in trans position. These compound heterozygous variants did not result in significantly increased Lyso-GL-1 level, the substrate level was only slightly elevated with 14.6 ng/mL (ref range: 0.0–14 ng/mL), and her enzyme activity was 5.8 μmol/L/h (cutoff value 3.3 μmol/L/h; Medical Laboratory, Archimed Laboratory Vienna, Austria). Hepatosplenomegaly was ruled out by abdominal ultrasound. Her sister carried the *GBA1* E365K variant, and her uncle was a carrier of the *GBA1* T408M and the SPG11 Q1875* variants. Neither of them had any signs of PD or other neurological symptoms.

#### Case 2

P16 was born prematurely at 7.5 months, but his psychomotor development was normal. He noticed occasional tremors in his hands from the age of 17, but it only became pronounced at the age of 31, when stiffness in the neck and the lower limbs occurred as well. At the early stage of his disease, DaTSCAN revealed mild dopamine transporter deficiency in the left striatal region. His handwriting changed, micrographia was observed, and bradykinesia had developed. During the next 15 years, tremor-dominant Parkinsonian symptoms developed with coexisting severe spasticity in the lower limbs. Dysdiadochokinesis, limb ataxia, and myoclonus appeared as well in the upper limbs. His cognition was not affected; however, he complains of mixing up words during conversation 15 years after the debut of his symptoms. Brain MRI detected only slight cerebellar atrophy. The results of his WES analysis identified the *GBA1*: c.882T>G/H294Q heterozygous and *SMPD1* (NM_000543.5): c.872G>A/R291H heterozygous rare variants. The *SMPD1* variant can be classified as a “variant of unknown significance” based on ACMG rules (PM1, PM2, PP2, PP5), in the ClinVar database it is classified as “conflicting interpretations of pathogenicity” with 1 pathogenic and ten VUS submission (variation ID: 195085). This patient’s ß-glucocerebrosidase enzyme activity was 2.8 μmol/L/h (cutoff value 1.5 μmol/L/h; Medical Laboratory, Archimed, Vienna, Austria), and acid sphingomyelinase enzyme activity was 1.6 μmol/L/h (cutoff value 1.2 μmol/L/h; Medical Laboratory, Archimed, Vienna, Austria). Although not considered to be pathogenic, both values were in the lower range of the spectrum.

In his family, his father had neurological symptoms, and the paternal grandfather and his brother had PD symptoms. His father’s (P24) genetic test identified only the *GBA1* H294Q mutation. His neurological symptoms started at the age of 65 with shuffling gait, auditory hallucinations, mild depression, and apathy. At age 71, he had resting tremor in the hands and mild rigidity in the arms, and his fast alternating movements started to slow down in the hands. His affective symptoms, apathy, and cognitive decline worsened. His Addenbrooke Cognitive test score was 76/100. The brain MR performed at the age of 70 showed bilateral small T2 hyperintensities. His clinical symptoms indicated mild Lewy Body Dementia.

#### Case 3

P25, a female patient started having resting antagonist tremor at the age of 29. A neurological examination detected a postural tremor in her left hand which was provoked by intention. In addition, mild dysdiadochokinesis was detected on the left side. She had mild truncal ataxia and positive lateropulsion. Her brain MR and DATSCAN showed no pathological changes. She has moderate anxiety, and stress is provoking her tremor. She is currently 33 years old. She harbored coexisting heterozygous *GBA1* c.1223C>T/T408M and the c.347T>C/M116T heterozygous rare variant in the S*NCA* gene (NM_000345.4). The *SNCA* rare variant can be classified as a “variant of unknown significance” based on ACMG rules (PM2, BP4), and it is not described in ClinVar. Based on a literature search done using the MasterMind platform, this variant was mentioned in one publication screening *SNCA* variants in the UK Biobank, although its clinical effect was not discussed (PMID: 33307186). The patient’s ß-glucocerebrosidase enzyme activity was reduced to 2.13 μmol/L/h (cutoff value 3.3 μmol/L/h; Metabolit Screening Laboratory of Department of Pediatrics, Semmelweis University). Her paternal grandfather showed signs of PD around the age of 40 years, and two of her paternal uncles had apathy.

### Cascade testings

In our cohort, among the *GBA1* RV carriers, 11 patients had at least one family member with known PD or susceptible PD. We sequenced the specific exons of *GBA1* among relatives of *GBA1* RV carriers in six cases. Relatives with *GBA1* RV are presented in Table 4. In the rest of the cases, cascade testing was not possible.

**Table 4:** Positive results of GBA1 variants cascade testing among family members

Patient ID	Relative harboring the *GBA1* rare variant	Age of relative at test	Symptomatic	Older relative in the family with probable PD^a^
P2	Daughter	47	No	N/A
	Son	44	No	
P7	Father	67	No	N/A
P8	P9 (brother)^b^	34	Yes	Uncle
P10	Sister	68	No	Uncle^c^
	Uncle	86	No	
P14	Son	27	No	2 grandparents and mother
P16	P24 (father)^b^	70	Yes	Paternal grandfather and his brother

^a^In these cases, there were no genetic tests done; these findings are based on the exploration of patients’ anamnestic data

^b^Some data of these families was partially presented in our earlier publication [30]

^c^The uncle carrying the same GBA1 rare variant was not the PD-affected family member

## Discussion

Here we present the first comprehensive study of Hungarian patients with *GBA1*-associated Parkinson’s disease. We found that in our Hungarian PD cohort, 15.2% of the patients were carriers for at least one rare variant in the *GBA1* gene. An earlier study in Hungary examining the frequencies of only three variants (R159W, N409S, and L483P) of the *GBA1* identified three individuals with heterozygous L483P in a cohort of 124 PD patients [32]. The carrier frequency of L483P was similar in our cohort, but we detected several further rare variants, drawing attention to the necessity of broad genetic testing instead of looking for certain specific variants. The frequency of *GBA1* carriers in the investigated Hungarian PD group was higher (15.2%) compared to the 5.8% in Serbian [33] and 8% in Poland [34], however, similar to the 14.7% of the combined Slavic PD cohort [35]. The frequency of GBA1 carriers could even be higher, considering the possibility of false negative results due to the use of short-read NGS sequencing. Some complex gene-pseudogene rearrangements that could occur can go unnoticed without specifically addressing these types of recombinant events using Sanger sequencing or long-read sequencing methods.

The average age of onset among carriers of *GBA1* rare variants was 44.6 + 12.3 years, which did not differ from the non-*GBA1*-associated PD cohort, which was 45.5 + 10.7 years. Previous studies reported that the age of onset when carrying *GBA1* RVs is usually earlier compared to sporadic PD [36, 37]. This was not observed in our cohort, where the AOO of sporadic PD was 44+11.7 years. The AOO of both *GBA1* carriers and non-carriers groups are considered to be EOPD. This could be due to two factors: the skewed reports and screenings of *GBA1* variants in earlier studies and the fact that many older patients (age over 60) showing signs of PD do not get tested for genetic alterations. This could also explain why the average AOO of our cohort was 44.4 years. There was also no difference between the AOO of carriers between the two most frequent variants, E365K and T408M, and the rest of the cohort.

Even though the disease onset and the associating symptoms were variable in our cohort, comparing the phenotypes of carriers of E365K or T408M did not show a difference from the carriers of the commonly called “severe mutations” N409S or L483P, in cognitive dysfunctions, psychiatric symptoms, or autonomic dysfunctions. This further proves the importance of the genotyping of the whole gene. Three subjects carried more than one heterozygous rare variant in a gene known to be associated with Parkinson’s disease. Even though they did not exhibit a clear, more severe phenotype than patients with a single heterozygous variant, they showed great heterogeneity in their age of pseudo-onset, family history, anamnesis, and presenting symptoms. This is consistent with the highly heterogeneous manifestation of pathological markers seen in carriers of mutations in the *GBA1* gene. We assume that this heterogeneity of patients’ phenotype could be driven by different genetic alterations, possibly due to oligogenic effects [30, 38, 22].

Along with the assumption that coexisting genetic alterations may act as modifying factors in the phenotype of *GBA1* RV carriers, we screened for ApoE alleles in this group. Our findings indicate that the ApoE ε4 allele did not significantly affect the age of onset or the presence of cognitive dysfunction in carriers compared to non-carriers. This seems to be contradictory to the literature [28, 29]; however, a possible explanation is our limited sample size and the lack of longitudinal data concerning the progression of most of the subjects’ phenotype.

Several studies have explored the association between lysosomal dysfunction and a-syn aggregation, leading to the hypothesis that LSD metabolites may play a role in the induction of synucleinopathy and the inhibition of autophagic-lysosomal pathways due to a-syn aggregates [4]. Some genetic variants in the Sphingomyelin phosphodiesterase 1 (*SMPD1*) gene for example, known for Niemann Pick disease (acid sphingomyelinase deficiency), have also been implicated in increasing the risk of PD [39, 40]. This is supported by findings in one of our patients, who carried not just the *GBA1* H294Q, but also the *SMPD1* R291H heterozygous variant as well. His hand tremor started at age 17, and his Parkinsonian signs developed very slowly. The co-existing cerebellar signs and spasticity in his lower limbs were peculiar. Unfortunately, there is no detailed phenotypic description of patients having PD and carrying SMPD1 mutation published in the literature. The patient’s clinical phenotype and onset strongly differed from his father’s, who carried only the *GBA1* H294Q variant. This supports the hypothesis that disruptions in the lysosomal pathways may play a role in the background of PD [41], and the possible oligogenic effect behind disease severity [42]. Furthermore, it emphasizes the importance of employing broader sequencing methods to investigate the presence of additional mutations among our *GBA1* patients to confirm any intergenic interactions and the possibility of an oligogenic effect.

One of our interesting findings was the difference in genotype-phenotype between P10 and P25. P25 had low enzyme activity, and her symptoms started with tremor in her 30s. Her paternal grandfather showed signs of EOPD as well. The α-syn accumulation is thought to begin years or even decades before the onset of motor symptoms in PD [43], so we can hypothesize that a bidirectional pathogenic link between a-syn and GCase could influence the mildly reduced enzyme activity [11]. In comparison, P10, who carried two *GBA1* heterozygous variants in trans position, had normal enzyme activity and Lyso-GL1 level and had an age of onset in her 50s. Although we identified two carriers in her family (at the ages of 69 and 87), neither of them presently exhibited any neurological symptoms. Both patients carried an additional heterozygous rare variant (P10 *SPG11*: p.Q1875*; P25 *SNCA*: p.M116T ) that could have an effect on their disease development, but neither of these variants has been associated with PD in the literature so far. *SPG11* is associated with the recessive hereditary spastic paraplegia 11, which can have a complex phenotype in some cases. Patients with *SPG11* homozygous mutations can have tremor or parkinsonism, mostly in the later stages of their disease, which are mostly L-dopa responsive [44, 45]. Despite this connection to parkinsonism, we have not found any reports or cases that describe a PD patient with a heterozygous rare variant in the *SPG11* gene. This may explain why the family member of P10, who harbored the *GBA1* T408M and *SPG11* Q1875* variants, showed no signs of PD nor hereditary spastic paraplegia. The existence of asymptomatic relatives carrying multiple rare variants should caution against declaring that these special cases are solely based on genetics, but it cannot be disputed that rare variants are a significant factor at the core of the disease. Stratifying *GBA1*-associated PD is important because it allows for a more personalized approach to treatment and care for patients. Different genotypes can result in varying disease courses and symptoms, as well as differing responses to treatment. Several clinical trials are ongoing for patients with GBA-associated PD: One trial, PROPEL, studies the effect of PR001, which is a gene-replacement therapy that uses adeno-associated virus 9 (AAV9) to deliver a functional copy of the *GBA1* gene to the brain intracisternal (clinicaltrials.gov: NCT04127578). The other clinical trials are investigating the effect of ambroxol (DUPARG-Ambroxol and AMBITIOUS), which appears to facilitate the refolding of the misfolded GCase enzyme; thus, it may have therapeutic potential (clinicaltrials.gov: NCT05287503, NCT05830396).

By understanding the specific genotype of a patient, healthcare providers can tailor their treatment plan to optimize outcomes and potentially improve quality of life. Additionally, stratifying *GBA1*-associated PD can aid in identifying family members who may also be at risk for developing the disease, allowing for earlier detection and intervention.

Our findings address a gap in the available genetic data from Central-Eastern Europe and emphasize the significance of wider genotyping in the clinical field. Furthermore, our study provides a foundation for future prospective investigations.

## Conclusion

*GBA1* variants were relatively common (15%) in our Hungarian Parkinson’s disease cohort, with the E365K and T408M variants being the most frequent. However, when these two rare variants co-occurred in patients in our cohort, they did not demonstrate any distinct features in the phenotype. The presence of other coexisting variants in genes such as *SMPD1* and *SNCA* was found to modify the phenotype, but no modifications were seen in cases with the ApoE ε4 allele. Our research highlights the potential implications of identifying *GBA1*-related PD in the early clinical phase, enabling personalized disease-modifying therapeutic options for patients. Further exploration of gene-gene interactions in clinical settings is a vital step, not just for understanding the pathomechanism of this disease, but for the development of treatments and for comprehensive genetic counselling of the affected families.

### Supplementary Information

ESM 1 (DOCX 11.8 kb)

## Data Availability

The datasets generated during and/or analyzed during the current study are not publicly available due to concerns regarding participant/patient anonymity but are available from the corresponding author on reasonable request.
