# Supplementary material for: GBA-associated Parkinson’s disease in Hungary: clinical features and genetic insights
Source: Neurol Sci. 2023 Dec 28;45(6):2671–9. doi: 10.1007/s10072-023-07213-w (PMC11082009; doi:10.1007/s10072-023-07213-w)
Supplement: Supplementary file 1 — (DOCX 11.8 kb) [file 10072_2023_7213_MOESM1_ESM.docx]

**Supplementery file 1:**

The detailed gene lists of used targeted NGS panel sequencing.

**1. Targeted NGS panel gene list: Number of sequenced patients n=51**

ATP1A3,ATP6AP2,ATXN2,BACE1,BIN1,C19ORF12,C21ORF2,C9ORF72,CASS4,CD2AP,CD33,CELF1,CFL1,CHCHD10,CHCHD2,CHMP2B,CLU,COASY,CP,CR1,CTSD,CSF1R,CST3,DCAF17,DCC,DCTN1,DNAJC13,DNAJC5,DNAJC6,DNAL4,DNMT1,EIF4G1,ELAVL1,EPHA1,EPHA4,ERBB4,FA2H,FBXO7,FIG4,FTL,FUS,GBA,GCH1,GIGYF2,GRN,HNRNPA1,HNRNPA2B1,HTRA2,ITM2B,LRRK2,MAPT,MARK2,MARK4,MATR3,MS4A4A,MS4A4E,MS4A6A,MS4A6E,NEFH,NEU1,NOTCH3,OPTN,PANK2,PARK2,PARK7,PFN1,PICALM,PINK1,PLA2G6,POLG,PRKRA,PRNP,PRPH,PSAP,PSEN1,PSEN2,PTK2B,RAD51,SCP2,SETX,SIGMAR1,SLC30A10,SLC6A3,SNCA,SNCB,SOD1,SORL1,SPG11,SPR,SQSTM1,SYNJ1,TAF1,TARDBP,TBK1,TBP,TFG,TH,THAP1,TMEM106B,TMEM230,TOMM40,TOR1A,TPP1,TREM2,TUBA4A,TYROBP,UBE3A,UBQLN2,UCHL1,UNC13A,VAPB,VCP,VPS13C,VPS35,WDR45

**2. Targeted NGS panel gene list: Number of sequenced patients n=87**

CP,SOD1,LRRK2,PLA2G6,PARK2,SPG11,PRNP,PSEN2,SPG7,PARK2,C19orf12,ATM,APP,SNCA,PINK1,CP,PARK7,GBA,SPAST,TREM2,CSF1R,MAPT,TARDBP,ATM,SPG11,PSEN1,GRN

**3. Whole exome sequencing: Number of sequenced patients n=14**

GBA1 positive patients underwent WES were P7, P14, P16
